# Supplementary material for: Effect of Omega 3 supplementation during pregnancy and lactation on cognitive functions in rat offspring
Source: PLoS One. 2025 Sep 19;20(9):e0319457. doi: 10.1371/journal.pone.0319457 (PMC12448978; doi:10.1371/journal.pone.0319457)
Supplement: Attachment — Submitted filename: Peer review.docx [file pone.0319457.s001.docx]

**Dear editor：**

After careful review of your manuscript investigating omega-3 supplementation in pregnant rats, I recommend substantial methodological enhancements to strengthen the scientific rigor and comprehensive interpretability of your research.

**Major Revisions Required**

1. Experimental Design Visualization:

Figure 1 should be redesigned as a comprehensive experimental workflow diagram.

Clearly illustrate: Omega-3 supplementation groups, Dosage stratification, Monitoring indicators, Precise observation timepoints.

Enhance visual clarity of experimental protocol.

2. Sample Size and Dosage Optimization:

Expand sample size to improve statistical power

Implement dosage gradient groups: Low dose: 200 mg/kg, Medium dose: 400 mg/kg, High dose: 600 mg/kg.

Conduct comprehensive dose-response analysis.

Ensure statistically significant group comparisons.

3. Gender-Comparative Cognitive Assessment:

Incorporate female offspring cognitive function evaluation

Perform Morris Water Maze tests for both male and female progeny.

Analyze potential gender-specific neurological responses.

Provide comprehensive sex-based comparative data.

4. Molecular Biological Indicators:

Recommended molecular markers

BDNF (Brain-Derived Neurotrophic Factor) protein levels

Phosphorylated CREB expression

5. Mechanistic Investigation of Omega-3 in Neural Development:

Elucidate potential molecular mechanisms

Neuroplasticity pathways

Synaptic development regulation

Potential epigenetic modifications

Propose comprehensive theoretical framework explaining omega-3's neurological impacts

**Supplementary Methodological Recommendations**

1.Standardize omega-3 source and purity

2.Detailed documentation of supplementation protocol

3.Comprehensive data presentation

These revisions will substantially enhance the manuscript's scientific contribution, providing a more nuanced understanding of omega-3's role in prenatal neural development.

Sincerely,

PLoS One Editorial Board
