# Supplementary material for: Effect of Omega 3 supplementation during pregnancy and lactation on cognitive functions in rat offspring
Source: PLoS One. 2025 Sep 19;20(9):e0319457. doi: 10.1371/journal.pone.0319457 (PMC12448978; doi:10.1371/journal.pone.0319457)
Supplement: Attachment — Submitted filename: Peer review_HNOO_16.05.2025.docx [file pone.0319457.s004.docx]

**Dear editor：**

After careful review of your manuscript investigating omega-3 supplementation in pregnant rats, I recommend substantial methodological enhancements to strengthen the scientific rigor and comprehensive interpretability of your research.

**Major Revisions Required**

1. Experimental Design Visualization:

Figure 1 should be redesigned as a comprehensive experimental workflow diagram.

Clearly illustrate: Omega-3 supplementation groups, Dosage stratification, Monitoring indicators, Precise observation timepoints.

Enhance visual clarity of experimental protocol.

**Answer**

**Figure 1 convert to Table 1 and added experimental protocol.**

2. Sample Size and Dosage Optimization:

Expand sample size to improve statistical power

Implement dosage gradient groups: Low dose: 200 mg/kg, Medium dose: 400 mg/kg, High dose: 600 mg/kg.

Conduct comprehensive dose-response analysis.

Ensure statistically significant group comparisons.

**Answer**

**Sample size is calculated by power analysis.**

**When deciding on the dose, it was desired to be close to the daily dose. For this reason, when the studies were examined, the decision was made to use 400 mg/kg/day, which is mostly given.**

**A single dose was administered based on the animals’ body weight, as the aim of the study was to investigate the effects of the duration of omega-3 supplementation rather than a dose-dependent response.**

3. Gender-Comparative Cognitive Assessment:

Incorporate female offspring cognitive function evaluation

Perform Morris Water Maze tests for both male and female progeny.

Analyze potential gender-specific neurological responses.

Provide comprehensive sex-based comparative data.

**Answer**

**Behavioral testing using the Morris Water Maze was conducted when the offspring reached postnatal day 45, an age considered sufficient for the development of spatial learning and memory abilities. To minimize the potential confounding effects of hormonal fluctuations associated with the estrous cycle, which typically begins between postnatal days 30 and 40 in female rats, only male offspring were included in the experimental procedures**

4. Molecular Biological Indicators:

Recommended molecular markers

BDNF (Brain-Derived Neurotrophic Factor) protein levels

Phosphorylated CREB expression

**Answer**

**We appreciate the reviewer’s valuable suggestion regarding the inclusion of molecular markers such as BDNF and phosphorylated CREB to enhance the mechanistic understanding of our findings. We fully agree that these markers could provide important insights into the neural mechanisms underlying cognitive function.**

**However, due to the financial and technical limitations of our current project, as well as the lack of access to molecular analysis facilities and expertise at the time of the study, we were unable to include these analyses within the scope of this research.**

**We acknowledge this limitation and have noted it in the Conclusion section of the revised manuscript. We also consider this an important direction for future studies that aim to explore the molecular underpinnings of omega-3 supplementation more deeply.**

5. Mechanistic Investigation of Omega-3 in Neural Development:

Elucidate potential molecular mechanisms

Neuroplasticity pathways

Synaptic development regulation

Potential epigenetic modifications

Propose comprehensive theoretical framework explaining omega-3's neurological impacts

**Answer**

**In accordance with the reviewer’s suggestion, an additional paragraph has been included to discuss the potential molecular mechanisms by which omega-3 supplementation may influence cognitive development. This section provides a theoretical framework addressing neuroplasticity, synaptic regulation, and epigenetic modifications**.

**Supplementary Methodological Recommendations**

1.Standardize omega-3 source and purity

**Answer**

**In our research we used Solgar Omega 3 950mg EPA+DHA. That is mentioned in the article.**

2.Detailed documentation of supplementation protocol.

**Answer**

**Table 1 is given the detail of the protocol.**

3.Comprehensive data presentation

**Answer**

**In response to the reviewer’s comment on comprehensive data presentation, the Methods section has been revised to include clearer descriptions of the experimental design, grouping, and supplementation protocols, ensuring transparency and reproducibility.**
