# Supplementary material for: Effect of Omega 3 supplementation during pregnancy and lactation on cognitive functions in rat offspring
Source: PLoS One. 2025 Sep 19;20(9):e0319457. doi: 10.1371/journal.pone.0319457 (PMC12448978; doi:10.1371/journal.pone.0319457)
Supplement: Attachment — Submitted filename: Peer review 7-9.docx [file pone.0319457.s003.docx]

**Dear editor：**

After carefully reviewing your manuscript on the supplementation of omega-3 in pregnant rats, I suggest modifying the layout details of the table and integrating and modifying the paragraphs of the written content to make the readability of the article more in line with the style of the journal and facilitate better understanding and joint discussion by readers.

**Minor Revisions**

1. In Table3, Table4, X̄±SS (s) is arranged in alignment with the results in the table, which is convenient for readers to read.

**2.** Remove the vertical lines from Table1 to maintain the same specifications as other Tables 2-5.

**Supplementary Methodological Recommendations**

1. Could the result paragraph be extracted separately for writing, and the results and conclusions be integrated into a single paragraph? This would be more in line with the style of the journal and more conducive to readers' reading and analysis.

These revisions will substantially enhance the manuscript's scientific contribution, facilitating better reading by readers and joint exploration of the effectiveness and reproducibility of omega-3.

Sincerely,

PLoS One Editorial Board
