# Supplementary material for: Effect of Omega 3 supplementation during pregnancy and lactation on cognitive functions in rat offspring
Source: PLoS One. 2025 Sep 19;20(9):e0319457. doi: 10.1371/journal.pone.0319457 (PMC12448978; doi:10.1371/journal.pone.0319457)
Supplement: Attachment — Submitted filename: Answers to Editoral Board and Reviewer#2_01.08.2025.docx [file pone.0319457.s005.docx]

**Answers to Editoral Board and Reviewer#2**

**Minor Revisions**

1. In Table3, Table4, X̄±SS (s) is arranged in alignment with the results in the table, which is convenient for readers to read.

*Answer: The values are presented as X̄ ± SD (s); however, visual misalignment may occur across cells since the number of characters is not uniform.*

2. Remove the vertical lines from Table1 to maintain the same specifications as other Tables 2-5.

*Answer: Table 1’s vertical lines was removed. Also table names are updated.*

**Supplementary Methodological Recommendations**

1. Could the result paragraph be extracted separately for writing, and the results and conclusions be integrated into a single paragraph? This would be more in line with the style of the journal and more conducive to readers' reading and analysis.

*Answer: The Results and Discussion sections were divided into separate parts, and the Conclusion section was shortened and refined to present a more succinct summary.*

**Answer for Reviewer#2**

The authors main claim is that supplementation of omega-3 supplementation either in utero or in the antenatal period could have positive impacts on cognition of offspring

It seems that this topic has been tested within the field and sources cited in the results and discussion seem to show that there is data on timing during pregnancy and supplementation

It is unclear to me how this study is novel from the studies cited. For example, in lines 353-363 studies are outlined that appear very similar to this one. I’m not sure the authors have clearly outlined their novelty to the field.

*Answer: In previous studies, supplementation was typically initiated during the late stages of pregnancy. In contrast, this study uniquely applied nutritional supplementation starting from the preconception period. This has been explicitly outlined in the aims section of the study.*

The introduction is confusing to read, and the authors should do some significant editing to direct the introduction in a way that makes their arguments clear and well supported. The discussion of epigenetics is confusing and does not align with the major goals of the paper.

*Answer: Since this study seeks evidence that not only genetic factors but also epigenetic factors such as nutrition and environmental influences may play a role in shaping physical and cognitive potential during early infancy, the inclusion of epigenetics is both relevant and necessary. To strengthen the conceptual link with the topic, a brief definition of epigenetics has been added.*

The first paragraph starts out with a focus on the first 1000 days of life but then the authors deviate and begin to talk about the preimplantation period. This is difficult to follow as a reader.

*Answer: The importance of the preconception period has been highlighted in the revised version.*

There are numerous areas where more citations are needed. It appears the authors are using factual information but are applying in-text citations minimally. For example, the sentence from lines 178-180 should have a citation to let the reader know which study this is derived from.

*Answer: The manuscript has been updated to include the appropriate references.*

The interpretation of the data is not sufficient as is.

1.) there are measurements being made regarding weight when food is being given ad libitum. Without controlled intake of food, how can one make any inference regarding weight gain due to treatment?

*Answer: Since the rats were pregnant, food was provided ad libitum. For the purposes of our study, the primary outcomes of interest were maternal weight gain during pregnancy and the number of live-born pups, rather than the exact amount of food consumed.*

2.) The goal was to investigate if timing and length of supplementation has an effect. I don’t see how this is clearly evaluated. The tables are missing legends to let the reader know what the superscripts indicate (e.g. why do some groups have vs. b, vs. a,c). Without this it’s difficult to discern where there are true differences in timing. As is, it appears that the treatment had minimal effects, with some moderate changes in timing early (on 1st and 2nd days for platform finding, but this is lost in later days.

*Answer: To improve the clarity of the experimental design Table 1 has been added and also legands added to tables. Although an increase in platform-finding performance was expected on the 3rd and 4th days, the lack of statistical significance in these results was unexpected.*

3.) There is emphasis on significant differences in finding the platform across all groups for the different trials but isn’t this to be expected?

*Answer: Statistically significant differences in platform-finding latency among groups across different trials are expected; however, identifying which groups exhibit shorter durations and higher success rates is crucial for interpreting the effectiveness of the intervention.*

4.) Given the probe test has no significance, how does that impact overall conclusions regarding cognition? There is only one sentence to address the concept of reference memory.

*Answer: In the discussion section it is mentioned that “Cognitive function tests (Open Field Test, Elevated Plus-Maze, and Barnes Circular Maze) conducted on the offspring of rats that received adequate or inadequate n-3 fatty acid supplementation during pregnancy found a statistically significant difference in long-term memory tests among the offspring of mothers who received sufficient n-3 fatty acid supplementation………………………………….This may be due to the possibility that a single test may not be sufficient to assess reference memory.”*

This paper requires major revisions both in structure and interpretation and data delivery:

1. The whole introduction needs to be more concise and focus on key aspects of the study (e.g.- why did they select these particular periods- what is already known during these periods?? This is brought up more in the discussion and that makes it seem as if they are answering questions that are already answered). There are a lot of redundant statements (e.g. the sentence from line 46-47 is unnecessary) and paragraphs that lack organization paragraph starting at line 74 is extremely long and combines epigenetics with non-epigenetic health issues).

*Answer: To enhance clarity and coherence, the mentioned sentences and paragraphs have been revised in accordance with the overall context of the study.*

2. Nomenclature needs to be corrected throughout the paper (e.g. latin terms should be italicized)

*Answer: Latin terms are italicized. Nomenclature is checked.*

3. The methods need major revision. Figure 1 is not useful. Is treatment started at the particular time points and continued for a specific amount of time? Did the pre-conception dams get treatment throughout pregnancy whereas the lactation animals only started postpartum for a specific window. This is very unclear. How can the estrous cycle impact results? This isn’t explained clearly. Why were only two males taken per dam?

*Answer: In the method section these suggestions is addressed.*

4. The results are confusing as shown. No legend for the tables to allow the reader to fully understand notation shown.

*Answer: Legends are added.*

5. The interpretation of results needs to be re-approached. While negative data can be useful, its unclear what their study as a whole tells us regarding supplementation and timing (which was the initial goal). The use of literature in the discussion is confusing where at times it seems they focus on results from another study more than their own. The first sentence of the conclusion makes it seem that they had a drastic change due to treatment, when that doesn’t seem apparent as is.

*Answer: We hope that separating the Results and Discussion sections has improved the clarity of the manuscript. The Conclusion section has also been revised to better reflect the study’s key findings.*
